# Adsorption of methyl violet dye onto a prepared bio-adsorbent from date seeds: isotherm, kinetics, and thermodynamic studies

**DOI:** 10.1016/j.heliyon.2022.e10276

**Published:** 2022-08-15

**Authors:** Nisreen S. Ali, Noor M. Jabbar, Saja M. Alardhi, Hasan Sh. Majdi, Talib M. Albayati

**Affiliations:** a Mustansiriyah University, College of Engineering, Materials Engineering Department, Baghdad, Iraq; b Biochemical Engineering Department, Al-Khwarizmi Engineering College, University of Baghdad, Baghdad, Iraq; c Nanotechnology and Advanced Materials Research Center, University of Technology, Iraq; d Chemical Engineering Department and Petroleum Industries, Al-Mustaqbal University College, Babylon 51001, Iraq; e Chemical Engineering Department, University of Technology- Iraq, 52 Alsinaa St., PO Box 35010, Baghdad, Iraq

**Keywords:** Wastewater treatment, Environmental pollution, Methyl violet dyes, Batch adsorption, Date seeds characterisations, Adsorption isotherms, Adsorption kinetics: bio adsorbent, Adsorption mechanism, Adsorption thermodynamic parameters, Natural adsorbent

## Abstract

Raw date seeds, as prospective natural, broadly obtainable and low-price agricultural waste for adsorbing cationic dyes from aqueous solutions, have been studied. In this work, Iraqi date seeds were prepared and characterised using X-ray diffraction (XRD), scanning electron microscopy (SEM), Fourier transform infrared (FTIR) spectroscopy and Brunauer–Emmett–Teller (BET) surface area analysis before being used as an efficient bio-adsorbent for methyl violet (MV) dye removal. Adsorption tests were conducted with three investigated parameters, namely, time of contact, first adsorbate concentration and adsorbent dose. Compared with the pseudo first-order model (coefficient of determination = 0.9001), the pseudo second-order model was determined to be the best-fitting model with a coefficient of determination (R^2^) of 0.9917. The equilibrium isotherms for MV were obtained, and their ultimate capacity of adsorption was (59.5 mg g^1^). Two isotherm models, Langmuir and Freundlich, were studied to fit the equilibrium data. Compared with the Freundlich isotherm model (R^2^ = 0.8154), the Langmuir model functioned better as an adsorption isotherm with R^2^ of 0.9837. In addition, the adsorption process was endothermic and spontaneous. The date seeds acted as active adsorbents to remove MV from the aqueous solutions in the model experiments.

## 1 Introduction

Organic dyes are widely used to colour final items in various industries, such as textiles, cosmetics, food, paper, rubber, plastics and pharmaceutical plastic. Around 70 million tons of fabricated dyes are manufactured yearly for the international textile industry [1], and approximately 10%–15% of fabricated dyes utilised in industries are dumped into the atmosphere, resulting in serious aquatic contamination [2]. These hazardous colorants affect the aquatic environment because they obstruct aquatic plants’ photosynthesis ability by blocking the penetration of light and enhancing toxicity; moreover, the majority of these dyes induce gene mutation, cancer, dermatitis and allergies [3].

Methyl violet 2B (MV), a cationic dye, is particularly essential due to its wide range of uses in paints, textiles and print inks [4]. Cotton, paper, silk, bamboo, leather and straw are all dyed using MV [5]. MV is the active component in Gram's biological stain for bacteria categorisation in biomedical disciplines [6]. It can be applied as a moderate-type disinfectant on occasion, but it is poisonous to most animals. The respiratory tract may be irritated by MV inhalation, and the gastrointestinal tract is usually irritated by MV intake [7].

Treating wastewater polluted with dyes is a tough and expensive task because dyes are produced from intricate aromatic structures that are resistant to microbial attacks, oxidising chemicals, heat, water and light [8]. The removal of effluents containing dyes with intrinsic toxicity is currently being given serious consideration. Different dye removal modes have been investigated to minimise their negative impacts on the environment [9]. These strategies include ozonation [10], coagulation–flocculation [11], bacterial treatment [12], electrochemical oxidation [13], photocatalytic oxidation [14], membrane filtration [15, 16], solvent extraction [17], biofilm utilisation [18] and adsorption [19, 20]. Amongst the methods of dye rejection, adsorption is the most frequently used and effective method that yields promising results [21] due to its design simplicity, wide range of applications and small number of dangerous secondary outputs [22]. It is widely used to treat wastewater contaminated by inorganic and organic hazardous chemicals because it does not need costly equipment and highly experienced workers [23].

Various low-cost adsorbents have been utilised for dye removal. In addition, various waste materials from agricultural and industrial activities, natural materials and bio-sorbents can be used as alternative adsorbents at a reduced cost [24]. Several researchers have recently indicated that the by-products/wastes of agriculture have the potential to be used as inexpensive sorbents in dye removal from wastewater. Different by-product wastes of agriculture can be used as adsorbents in the treatment of wastewater is presented in Table 1.

**Table 1:** Applications of agricultural by-product wastes as adsorbents for wastewater treatment.

No.	Adsorbents	Pollutant	Concentration of dye used (mg/l)	q_max_ mg/g	Ref.
1	Walnut sawdust	Methylene Blue	750	59.17	[25]
2	Rice husk	Methylene Blue	100	40.6	[26]
3	Mangrove plant leaf powder	Crystal Violet	----	200	[27]
4	Mangrove plant fruit powder	Crystal Violet	----	250	[27]
5	Almond peel	Methylene Blue	100	77–118	[28]
6	Spent tea leaves	Basic Violet 10	50	71.4	[29]
7	Raw coffee residue	Remazol Blue	500	179	[30]
8	Pine cone	Acid Blue 7	50	37.4	[31]
9	Spent tea leaves	Malachite Green	----	227.3	[32]
10	Wood apple shell	Methylene Blue	200	95.2	[33]

This work was included the implementing of a low cost bio-waste material such as; Iraqi Date Seeds powder as an efficient adsorbent for removal of Methyl Violet Dye from domestic wastewater in a batch adsorption system. The Date Seeds were manufactured from available locally material that is able to reuse in order to remove the environmental pollution with using the low price, and environmentally friendly bio-adsorbent material.

Date seeds, a waste product, were employed as a bio-adsorbent in this study to remove MV dye from an aqueous solution by a batch adsorption experiments under various experimental settings due to their availability as a waste resource. X-ray diffraction (XRD), scanning electron microscopy (SEM), energy-dispersive spectroscopy (EDAX), Fourier transform infrared (FTIR) spectroscopy and Brunauer–Emmett–Teller (BET) surface area were used to analyse the date seed adsorbents. Freundlich and Langmuir adsorption isotherms were also examined to identify the most suitable isotherm data. The adsorption process’ kinetics was likewise studied.

## 2 Materials and methods

### 2.1 Used materials

#### 2.1.1 Adsorbent preparation

Without any chemical treatment, date seed powder was employed directly for adsorption studies. Date seeds were pulverised in a steel mill to grain size <300 μm and dried for 24 h at 100 °C before being utilised as a bio-adsorbent.

#### 2.1.2 Adsorbate

**Figure 1:**
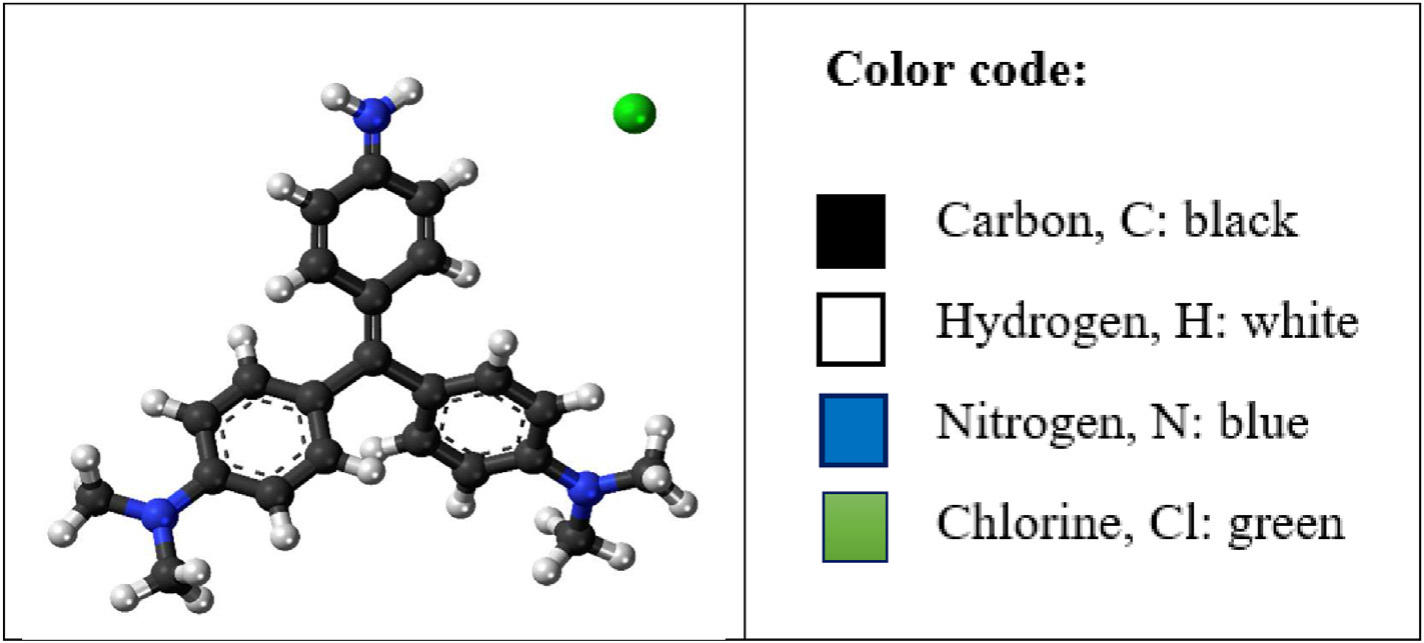
Structure of Methyl violet 2B dye.

MV 2B (molecular formula C_24_H_28_N_3_Cl, λ_max_ = 584 nm) dye was supplied by Germany Origin (Riedel–de Haen). The dye's structure is shown in Figure 1. The dye concentration in solution was measured with a spectrophotometer (UV-160 A, Shimadzu).

### 2.2 Methods

#### 2.2.1 Adsorption experiments

A variable amount of the adsorbent (0.5, 1, 3 and 5 g) was supplemented to (50 ml) of the dye solution with various concentrations (20, 40, 60 and 80 mg/l) then placed in (250 ml) conical flasks for batch adsorption experiments. All tests were conducted at room temperature (25 °C). The dye concentration in the solution beyond the equilibrium adsorption was obtained spectrophotometrically at λ_max_ = 584 nm for MV dye. After adsorption, the solution was centrifuged for 15 min at 3,000 rpm to extract the remaining dye concentration. Aqueous solutions of HCl or NaOH was used to modify the adsorbate solution pH. The removal of the dye from the solution was calculated as [34, 35]:

(1) $$\%\;Removal=\frac{C_{o}-C_{e}}{C_{o}}\times 100.$$

The adsorption capacity, *q*_e_ (mg·g^−1^), was estimated as [36, 37].

(2) $$q_{e}=\frac{\left(C_{o}-C_{e}\right)V}{M}\;,\;$$

where *C*_*o*_ and *C*_*e*_: first and equilibrium concentrations (mg·L^−1^) of the adsorbate, respectively; V: solution volume (L); M: adsorbent mass (g); qe: adsorbed amount (mg·g^−1^).

### 2.3 Characterisation

The crystalline structure of the date seed samples was determined using an XRD instrument (XRD-6000, Shimadzu, Japan), which produced radiation with a wavelength of 0.15405 nm. The system worked at a current of 80 mA and a voltage of 60 kV. SEM was employed to determine the sample composition, and EDAX was conducted to qualitatively record the element composition (Tescan VEGA 3 SB, SEM). FTIR (8400S, Shimadzu, Japan) was also used for the characterisation of the surface chemistry of the samples. The samples specific surface area of were checked using a Brunauer–Emmett–Teller device (type: Q-surf 9600, origin: USA).

### 2.4 Kinetic and isotherm studies

To calculate kinetic parameters for dye adsorption, tests were conducted with (5 g) of date seeds for MV solution with (50 mL) of (20 mg L^−1^) dye solution. The dye concentration was determined after 60, 90 and 120 min at room temperature. Moreover, 5 g of date seeds were used in the equilibrium adsorption studies with (50 mL) of (20–80 mg L^−1^) of the MV solution at room temperature. The concentrations of the dye solution before and after adsorption were assessed spectrophotometrically at (584 nm) wavelengths for the MV dye in all of the above-mentioned tests. The kinetic pseudo first-order and second-order models used to describe the MV sorption kinetics on the date seeds are shown in Table 2.

**Table 2:** Adsorption kinetics and isotherms.

Type of Models		Equations	Parameters	Ref.
Adsorption Kinetics Models	pseudo-first-order	$\text{ ​log}\left(q_{e}-q_{t}\right)=\;\log\;q_{e}-\frac{K_{1}}{2.303}\;t$	q_t_ (mg·g ^−1^): removed amount of MV at time t. qe (mg·g ^−1^): equilibrium adsorption uptake K_1_ (min^−1^): rate constant of the first-order adsorption.	[38]
	pseudo-second-order	$\frac{t}{q_{t}}=\;\frac{1}{K_{2}\;{q_{e}}^{2}}+\;\frac{1}{q_{e}}\;t\;$	K_2_ (g mg^−1^ min^−1^): rate constant of the second-order adsorption	[39]
Adsorption Isotherm Models	Langmuir	$\frac{C_{e}}{q_{e}}=\frac{1}{q_{max}}C_{e}+\frac{1}{q_{\max}b}$	C_e_ (mg·L ^−1^): equilibrium concentration of the MV in the solution q_e_ (mg·g ^−1^): removed amount of MV at equilibrium. q_max_ (mg·g ^−1^): maximum adsorption capacity, b (L·mg^−1^): Langmuir constant	[40]
	Freundlich	$\ln q_{e}=\ln\;K_{f}+\frac{1}{n}\ln\;Ce$	K_F_ (mg/g)/(mg/L)^1/n^: MV adsorption capacity. n: heterogeneity factor.	[41]

## 3 Results and discussion

### 3.1 Material characterisation

**Figure 2:**
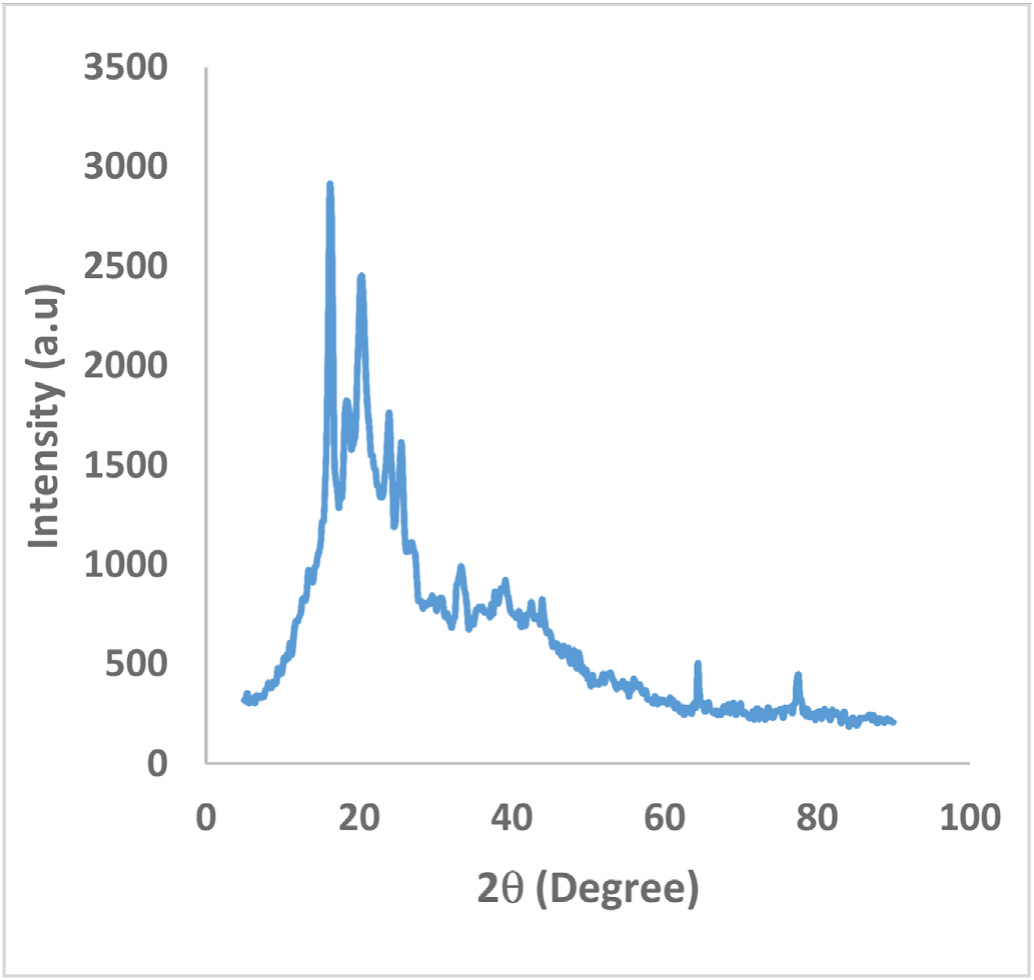
XRD analysis of date seeds.

**Figure 3:**
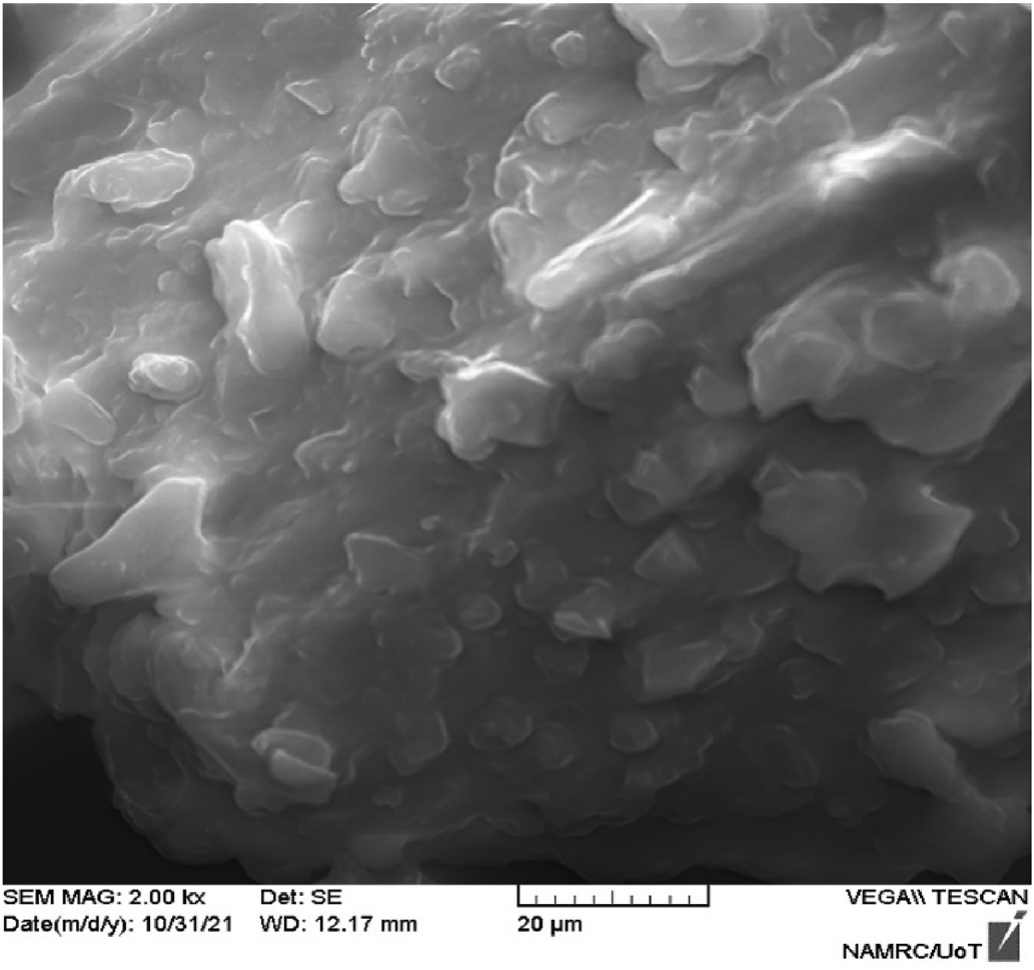
SEM image of the date seed structure.

XRD, SEM/EDAX and FTIR techniques were used to evaluate the pure adsorbent's phase purity and chemical and structural nature [42]. Figure 2 shows the XRD of date seed data. No horizontal basic line could be seen on the diffractogram, indicating the existence of diffraction peaks. This study revealed that the raw date pits had an amorphous primary structure with a low crystalline substance content. Given that adsorption is a physical phenomenon, it is mostly determined by surface properties, such as the number of holes on the adsorbent material's surface [43]. SEM was used to examine the adsorbent material's surface appearance and structure, as shown in Figure 3. The surface of the date seeds was rough and had some macropores according to the SEM examination. Furthermore, sufficient voids were present between date seed particles, which might have played a considerable role in the ion exchange and appropriate interaction between the date seed adsorbent and MV dye adsorption [44].

**Figure 4:**
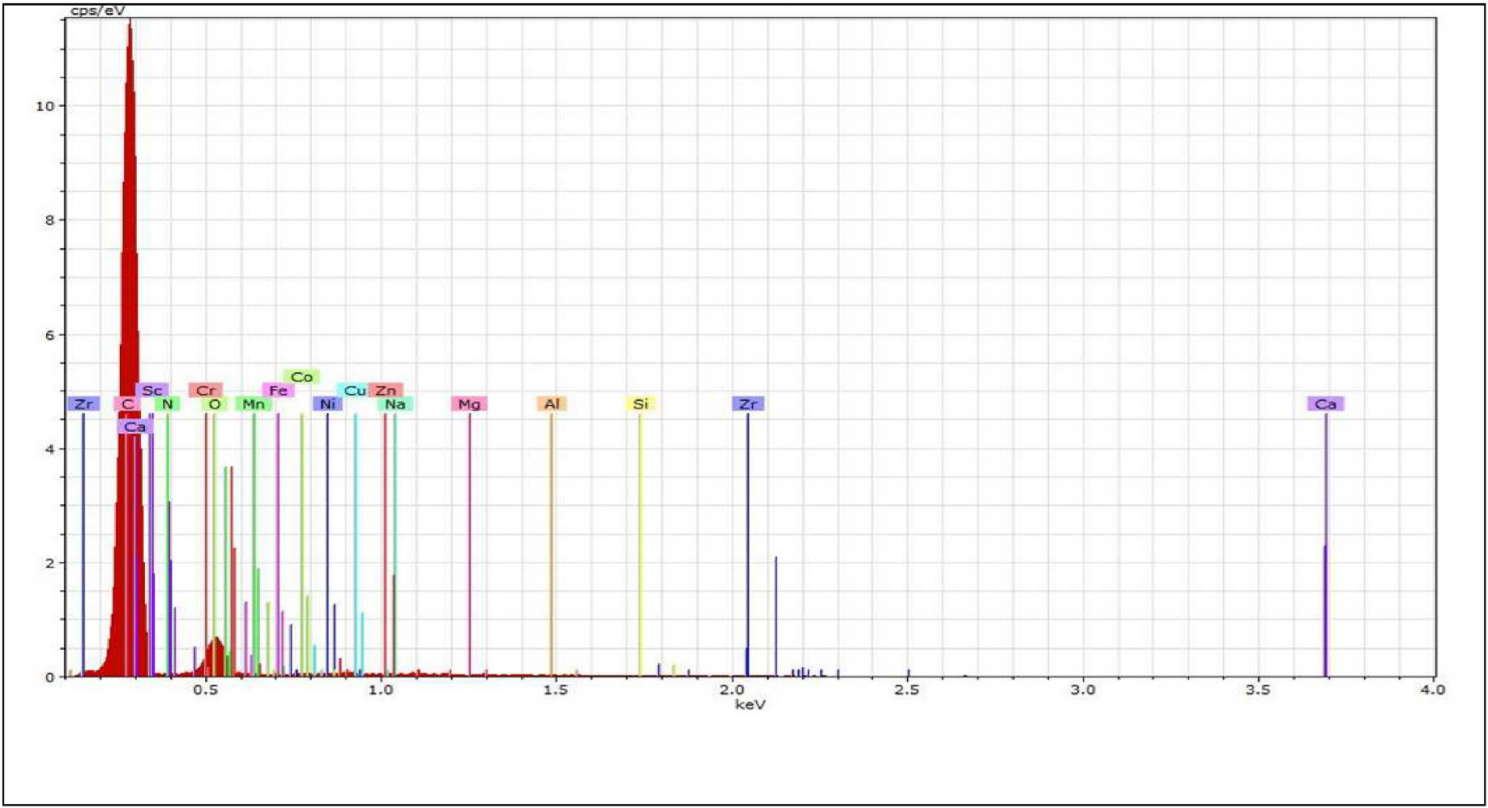
EDAX micrographs of the date seed.

EDAX, which was attached to the SEM instrument utilised to examine the bio-adsorbent morphology, was also used to evaluate the chemical composition of the date seed bio-adsorbent. The technique was used to measure the chemical species on the adsorbent surface. The EDAX analysis of fresh DS (Figure 4) provided a description of the elemental composition of the sample and the distribution of the elements. An important finding from this analysis was that the weight of metals expressed in percentage value as detected by EDX was in the following order: calcium (Ca), carbon (C) and oxygen (O), which were expectedly detected in the date seeds with values of 74.32%, 19.77% and 4.31%, respectively. The rest was distributed as follows: 0.69% chromium (Cr), 0.51% manganese (Mn), 0.14% nickel (Ni), 0.09% copper (Cu), 0.07% iron (Fe), 0.06% silicon (Si), 0.04% aluminium (Al) and 0.01% sodium (Na) (Table 3).

**Table 3:** The chemical composition of date seed.

Constituent	Concentration (wt.%)
Ca	74.32
C	19.77
O	4.31
Cr	0.69
Mn	0.51
Ni	0.14
Cu	0.09
Fe	0.07
Si	0.06
Al	0.04
Na	0.01
---	Total = 100

**Figure 5:**
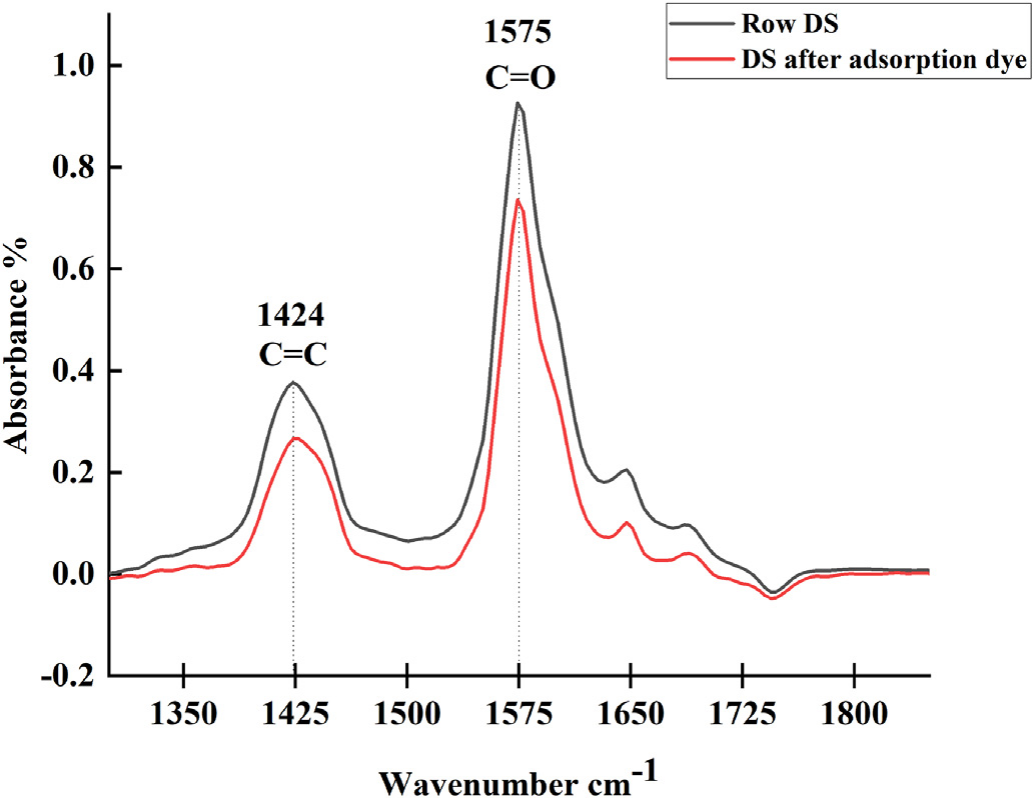
FTIR spectra of date seeds (DS) before and after adsorption of MV dye.

FTIR spectroscopy is a simple technique to quickly obtain information on chemical structures. In this study, FTIR was used to identify the chemical bonds and show how the MV dye affected the bonds. The FTIR spectra of the specimen before and after adsorption are shown in Figure 5. The FTIR spectra of the pure specimen show that the peaks of C=O (∼1500 cm^−1^) and C=C (∼1400 cm^−1^) had a higher intensity compared with those of the specimen after treatment [45, 46].

The FTIR spectra of the date seeds after MV dye adsorption back to the interaction between the positively charged MV dye molecules and the negatively charged date seed surface. This result proves that all of active adsorption sites on the date seed sample were consumed during dye adsorption [47]. The BET surface area and pore volume of the raw date seed sample were measured, and the results were 1.2 m^2^ g^−1^ and 0.02 cm^3^ g^−1^, respectively.

### 3.2 Effect of adsorbent dosage

**Figure 6:**
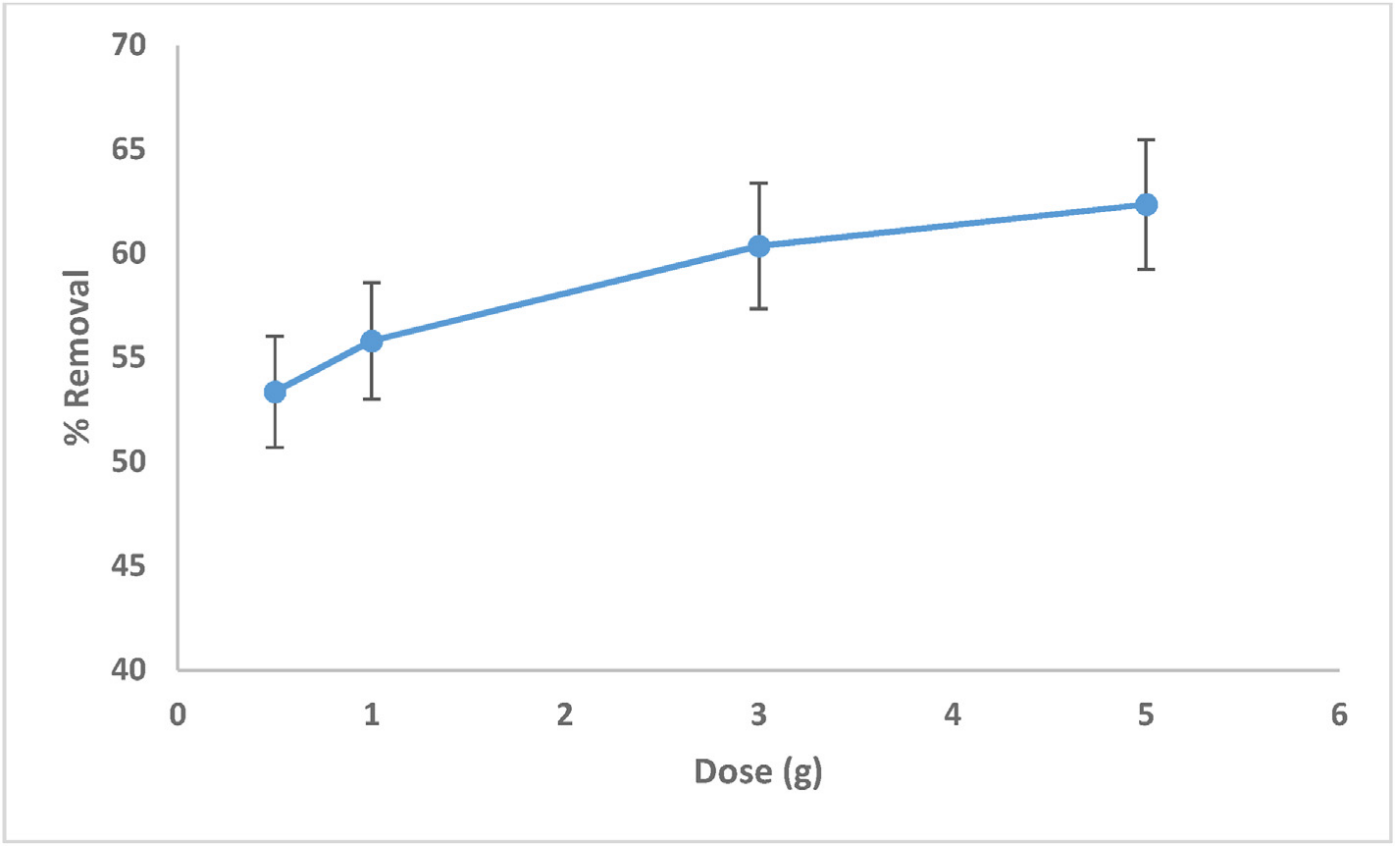
Effect of adsorbent dosage and MV dye removal using date seed (pH = 6.5, 25^0^C temperature, 120 min and 20 mg/L initial concentration of the dye).

The amount of the adsorbent is a crucial variable because it permits the maximum adsorption for a certain first concentration of the adsorbate to be calculated [48]. Figure 6 depicts the effects of cationic MV dye adsorbent dose. According to the findings from Eq. (1), increasing the dosage of the adsorbent caused an increment in the effectiveness of adsorption from (53.35%) to (62.35%). This result could be attributed to the increment in active adsorption locations and the adequate adsorbent surfaces as a result of increasing the adsorbent dosage [49].

### 3.3 Effects of initial concentration and contact time

**Figure 7:**
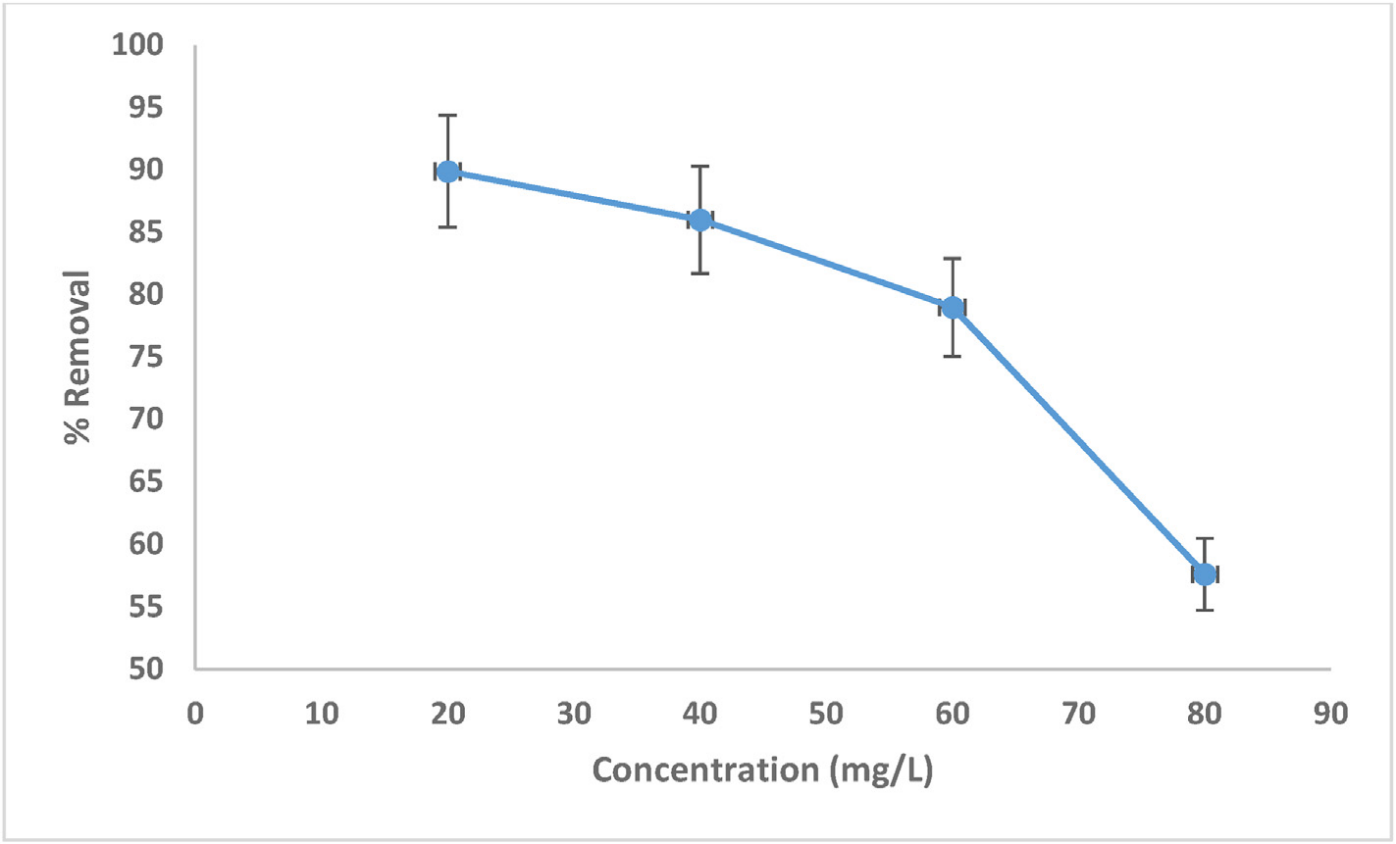
Effect of initial concentration of MV dye removal using date seed (pH = 6.5, 5 g adsorbent dosage and 120 min).

According to the findings from Eq. (1), the effects of the primary MV concentrations (20, 40, 60 and 80 mg/L) and contact period (60–120 min) on bio-adsorbent dye removal are shown in Figure 7. When the primary MV dye concentration increased from (20 mg L^−1^) to (80 mg L^−1^), the removal effectiveness decreased from 89.9% to 57.6%, respectively. In general, when the primary concentration of a dye in a solution increases, the locations of adsorption on the adsorbent surface become saturated, resulting in reduced removal effectiveness [50]. According to the findings from Eq. (2). The values of *q*_e_ and the mass transfer driving force increase because the MV dye molecules that surround the active sites of date seed increase as the initial concentration of MV dye increases [51].

**Figure 8:**
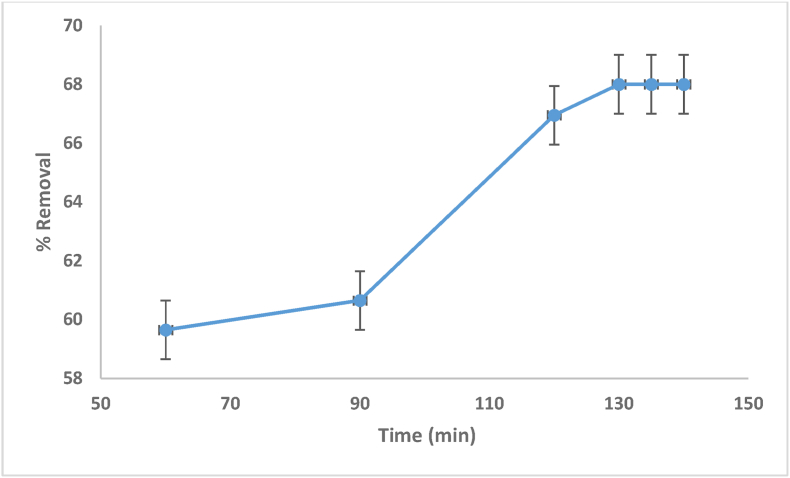
Effect of contact time on MV dye removal using date seed (pH = 6.5, 5 g adsorbent dosage and 20 mg/L initial concentration of the dye).

According to the findings from Eq. (1), allowing enough contact time to ensure that the system of the bio-sorbent dye has attained steadiness, after which no net mass transfer occurs between the phases of the solid and solution, is critical. It is also critical in the treatment of adsorption water/wastewater because the efficiency depends on quick bio-sorbent uptake and the development of steadiness in a short time [52]. Within the first 120 min in this study, MV was rapidly adsorbed (Figure 8). The process slowed down when the unoccupied locations on the bio-sorbent were occupied by the dye, and equilibrium was subsequently attained. Furthermore, after permitting the optimal length of shaking, the elimination extent at the steady state was not time dependent, so the period of settling of (2 h) was deemed optimal in all the steadiness experiments.

### 3.4 Adsorption kinetics and isotherm studies

**Figure 9:**
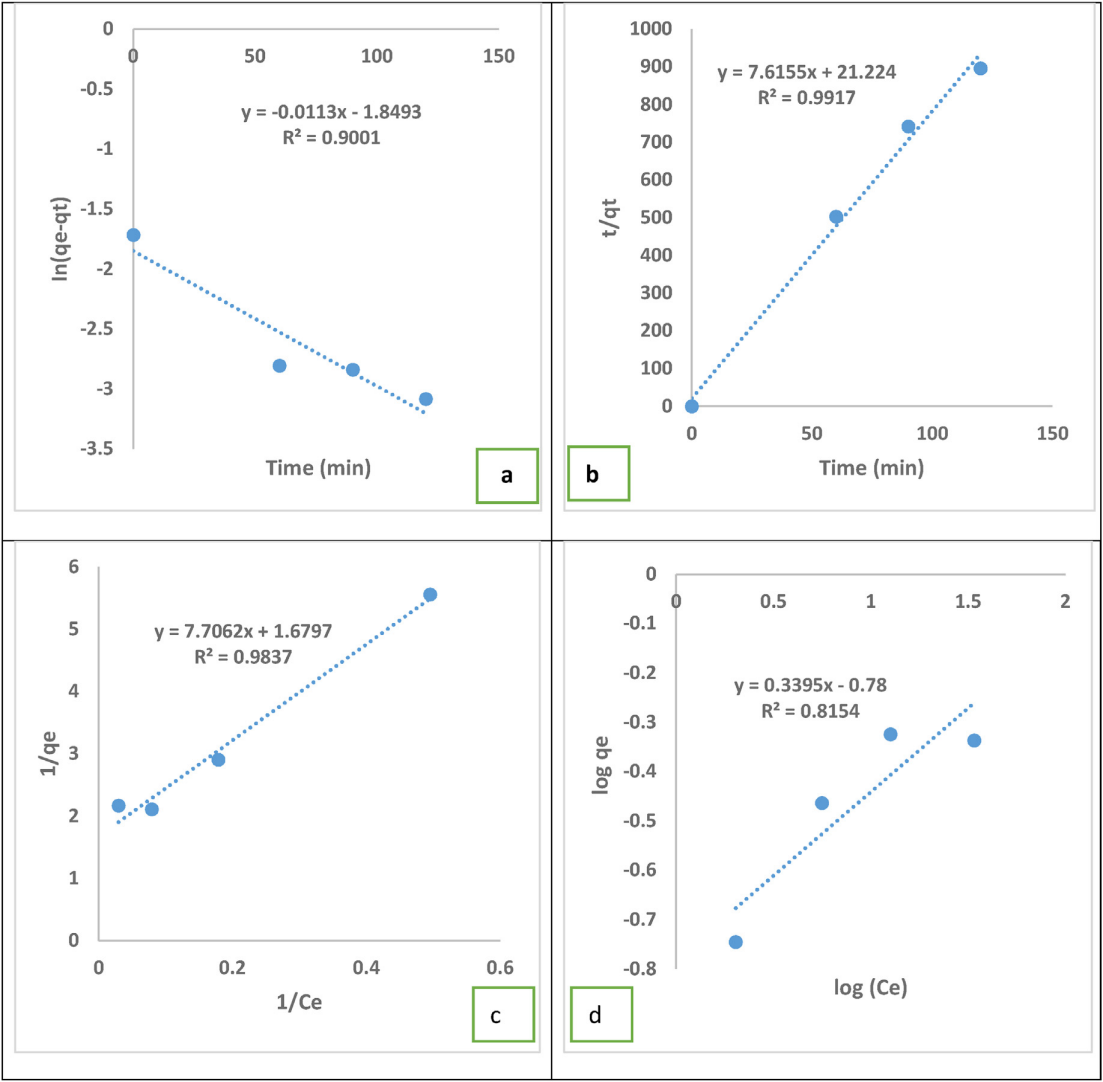
Kinetic and isotherm studies for MV dye adsorption on date seeds. (a) Pseudo-first order kinetics, (b) Pseudo-second order kinetics, (c) Langmuir Isotherm and (d) Freundlich isotherm.

According to the findings from Eq. (2) and with an R^2^ value of 0.9919, the pseudo second-order model provided the best depiction of MV sorption kinetics on the date seeds between the two kinetic models utilised in this investigation (Figures 9a and 9b; Tables 2 and 4). The use of pseudo second-order kinetics meant that the dye molecules’ chemical interactions with the adsorbent surface involved electron transfers. To attain the equilibrium concentration, the first phase consisted of quick chemisorption of the dye molecules on the date seed adsorbent, followed by the second slow phase of physisorption [53].

**Table 4:** Kinetic models for the sorption of MV dye onto date seeds.

Kinetic Models					
Pseudo-first order			Pseudo-second order		
q_e_ (mg/g)	K_1_ (min^−1^)	R^2^	q_e_ (mg/g)	K_2_ (g mg^1^ min^−1^)	R^2^
6.35	9.42E-05	0.9001	0.13	7.61	0.9917

Experiments were carried out with 5 g of adsorbent load and an initial feed concentration of 20, 40, 60 and 80 mg/L Figures 9c and 9d and Table 5 show the adsorption isotherm analysis’ outcomes. The theoretical ultimate capacity of adsorption (qmax) for MV dye uptake by date seeds was calculated as 59.5 mg/g using the Langmuir model. For dye adsorption on the adsorbent surface, the separation factor (R_L_) was equivalent to 0.043 (1), indicating good and spontaneous adsorption [53]. According to the comparison of the determination coefficient (R^2^) values for the two isotherm models, the equilibrium adsorption behaviour of the date seeds followed the Langmuir isotherm.

**Table 5:** Parameters of isotherm models of MV dye onto date seeds.

Isotherm Models						
Langmuir				Freundlich		
q_max_	R_L_	B	R^2^	*K*_*f*_	1/n	R^2^
0.595	0.043	0.217	0.9837	6.025	0.339	0.8154

### 3.5 Adsorption mechanism

The transfer of a solute from a solution to the adsorbent is described by four primary mechanisms. When the adsorbent is added to the solution, the initial step is the mass transfer of solute particles**.** This process is not considered when designing kinetic systems because it is too quick. Film diffusion is the second process, and it involves the gradual transport of solutes from the boundary layer to the surface of the adsorbent. The third mechanism is when the solute reaches the adsorbent's surface, and it moves to the pores. The final mechanism includes quick adsorptive adhesion of the solute to the pores active sites; because this is a fast process, and not interested during kinetics engineering design.

Film diffusion is the rate-controlling phase when the system has small solute size, poor mixing, and low concentration; the opposite of that, the process controlled by intraparticle diffusion (IP). The pseudo second-order model best explains the adsorption mechanism, when the solute concentration is low, however, when the initial concentration is high, the pseudo first-order model is preferred. This is because at low C_0_, the value of ln (qe−qt) rises exponentially, thereby increasing the error function, whereas at high C_0_, the value of ln (qe−qt) decreases exponentially, thereby decreasing the error function [54, 55, 56].

### 3.6 Effect of temperature and thermodynamic parameters

**Figure 10:**
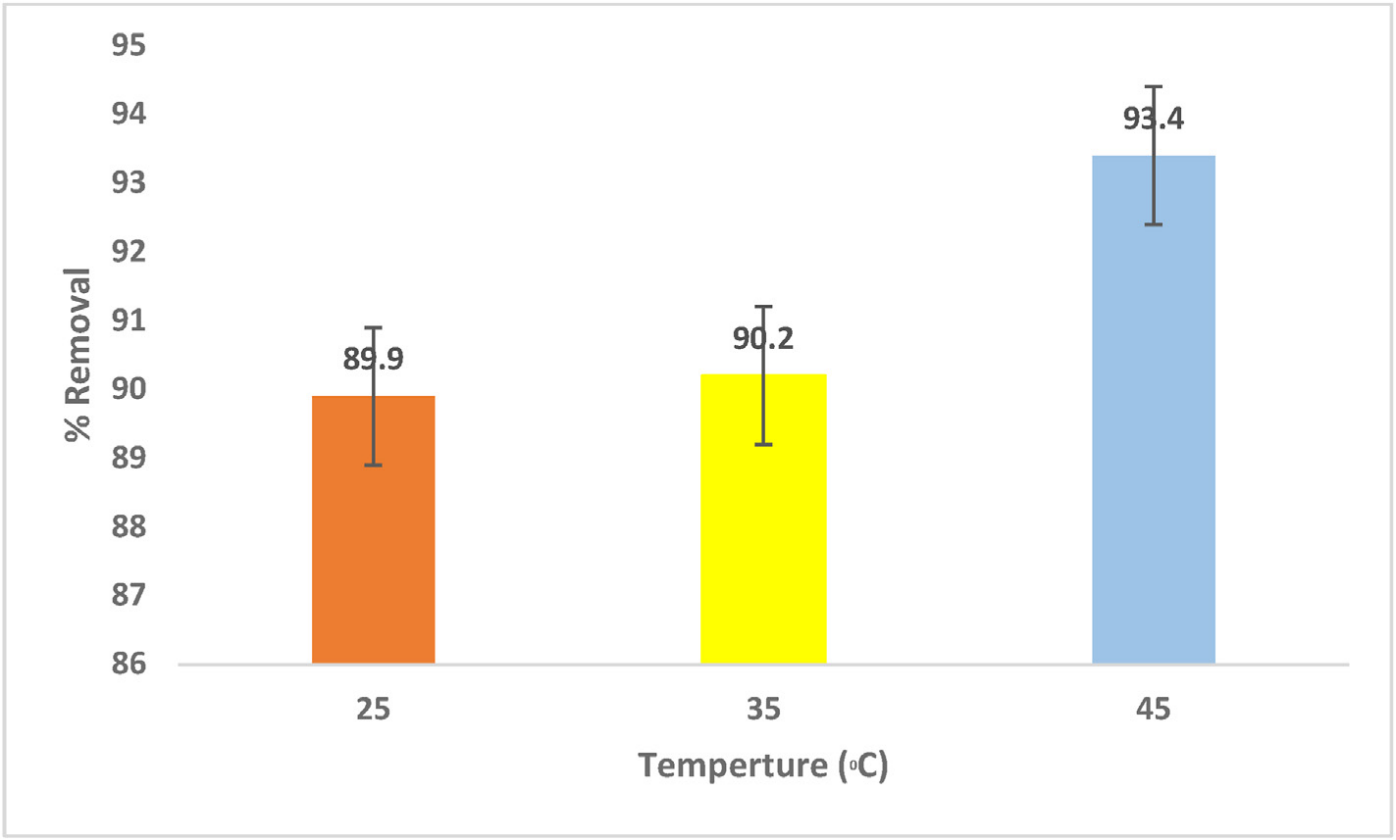
Effect of Temperature on MV removal (pH = 6.5, 0.5 g adsorbent dosage and 20 mg/L initial concentration of the dye).

The reduction of MV was investigated at various temperatures, including 25 °C, 35 °C and 45 °C, to determine the adsorption thermodynamic parameters. According to the experiment results, dye removal increased from 89.9% to 93.4% when the temperature rose from 25 °C to 45 °C, as shown in Figure 10. Changes in temperature affect the adsorbent's equilibrium capacity for a particular adsorbate because the dye molecules diffusion rate is a temperature-controlled process. In the current study, raising the temperature allowed the dye molecules to diffuse rapidly towards the external boundary layer and the adsorbent particles inner pores because viscous forces in the solution provided less resistance. In some conditions, the adsorbate molecules' solubility was also changed, which had a substantial impact on the removal process. Pore size enlargement may also be responsible for the rise in the adsorption capabilities of adsorbents at high temperatures [57, 58].

Thermodynamic studies are another essential aspect of adsorption studies. Thermodynamic metrics, namely, entropy change (S^o^), enthalpy change (H^o^) and Gibbs free energy (G^o^), were used in this study to assess the process’ spontaneous nature and thermodynamic feasibility. The following are the equations:

Gibbs free energy change (J/mol)):

(3) $$\Delta Gº=-RTlnK_{C},\;$$

where (Kc) is the apparent adsorption equilibrium constant given as [59]

(4) $$K_{C}=\frac{q_{e}}{C_{e}}.$$

In this case, the activity should be used instead of the concentration to obtain the adsorption system's standard thermodynamic equilibrium constant (*Kc*).

(5) ΔG^o^ = ΔH^o^–TΔS^o^

Entropy and enthalpy have a relationship that is described by [60]

(6) $$lnK_{C}=\frac{\Delta S{}^{\circ}}{R}-\frac{\Delta H{}^{\circ}}{RT}.$$

The slope and intercept of the Van't Hoff scheme of *ln K*_*c*_ versus 1/T (Eq. (6)) is used to obtain ΔH^o^ (J/mol) and *ΔS*^*o*^ (J/mol.K).

**Figure 11:**
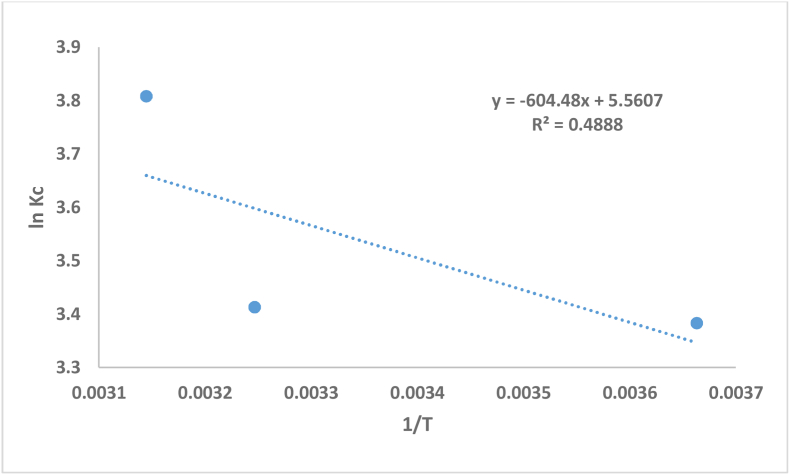
ln Kc vs 1/T plot for the adsorption of MV by 0.5 g DS (Co = 20 mg/l, pH = 6.5).

According to the findings from Eq. (4), the plot of the linear Van't Hoff equation (*ln Kc* vs. 1/T), shown in Figure 11, provides the values of thermodynamic parameters for the adsorption of MV on DS at different temperatures. The values are listed in Table 6. The table shows the experimental results of thermodynamic parameters for MV adsorption using DS. The positive value of (Δ*H*°= 5.0249 kJ/mol) According to the findings from Eq. (3) assures that the adsorption of MV onto DS is an endothermic reaction.

**Table 6:** Thermodynamic parameters for the adsorption of MV onto DS.

Temp. K	*ΔH*^*o*^ (KJ/mol)	*ΔG*^*o*^ (J/mol)	*ΔS*^*o*^ (J/mol.K)
298	5.0249	−7678.19	46.23
308		−8739.79	
318		−10068.7	

The adsorption process’ feasibility and spontaneity are manifested by the negative value of ΔG^o^. According to the findings from Eq. (5), the value of ΔS^o^ has been calculated to be extremely high, indicating a rise in entropy due to adsorption. The dyes ions on the surface of adsorbent are in a more ordered state before adsorption than in the following adsorbed condition, and the free dye ions ratio to interacting dye ions with the adsorbent is higher before adsorption than in the adsorbed condition. Therefore, the rotational distribution and translational energy increases as the adsorption increases, resulting in a positive value of entropy and enhanced randomness at the solid–liquid interface. Adsorption is more probable to happen spontaneously at high temperatures because ΔH^o^ > 0 and ΔS^o^ > 0 (Table 6) [57].

### 3.7 Batch regeneration system

**Figure 12:**
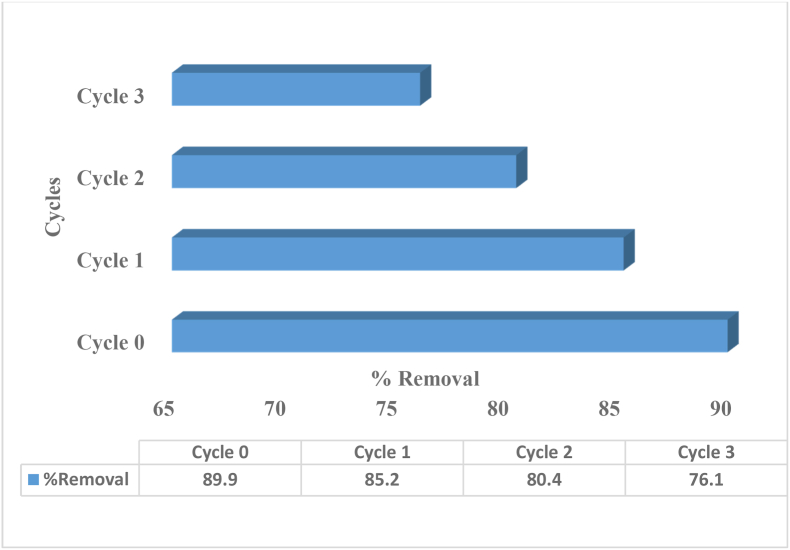
Reusability of DS in batch experiment (pH = 6.5, adsorbent dose = 0.5 g, contact time = 30 min, concentration of dye = 20 mg/L).

To lower the overall cost of the dye removal process, the used bio-adsorbent should exhibit effective extraction and recyclability during numerous adsorption operations. Hence, before considering practical applications, the regeneration of the bio-adsorbent should be considered. Date seed as a bio-adsorbent was shown to have scientific properties, such as durability, recyclability and high performance, in this investigation. The experiments were carried out numerous times using 0.3 mol. L^−1^ HNO_3_ and a liquid exchange method to liberate the adsorbed dye molecules and generate an MV-free bio-adsorbent. The treated date seed bio-adsorbent was filtered, dried and employed in a series of target molecule adsorption/extraction experiments. During the batch-contact process, the bio-adsorbent–dye solid was treated with 0.3 mol. L^−1^ HNO_3_ to liberate MV dye as a function of reuse/cycle numbers. Figure 12 shows evidence that the date seed bio-adsorbent may be recycled via de-complexation of the bound target molecules [61].

## 4 Conclusions

Raw date seed's ability to remove MV dye from a model water solution was investigated in this work. The Langmuir isotherm model with an ultimate biosorption capacity (qmax) of (59.5 mg g^−1^) was found to be correlated well with the experimental results obtained when the adsorption equilibrium was established by permitting (1 h) under ambient pH conditions. Furthermore, the pseudo second-order kinetics of MV sorption on date seeds was observed with a rate constant of (7.61 g/mg min). Thermodynamic studies confirmed that the adsorption method is endothermic and spontaneous. Despite the fact that raw date seeds have a smaller adsorption capacity than commercial activated carbon, raw date seeds are a cost-effective alternative adsorbent. The use of date seeds to remove colour from water could provide a cheap and effective adsorbent.

## Declarations

### Author contribution

Nisreen S. Ali, Noor M. Jabbar, Saja M. Alardhi: Performed the experiments; Contributed reagents, materials, analysis tools or data. Hasan Sh. Majdi: Conceived and designed the experiments; Performed the experiments. Talib M. Albayati: Conceived and designed the experiments; Performed the experiments; Analyzed and interpreted the data; Contributed reagents, materials, analysis tools or data; Wrote the paper.

### Funding

This research did not receive any specific grant from funding agencies in the public, commercial, or not-for-profit sectors.

### Data availability statement

All relevant data are included in the paper.

### Declaration of interest

The authors declare no conflict of interest.

### Additional information

No additional information is available for this paper.
